# A real-world evaluation of the diagnostic accuracy of radiologists using positive predictive values verified from deep learning and natural language processing chest algorithms deployed retrospectively

**DOI:** 10.1093/bjro/tzad009

**Published:** 2023-12-12

**Authors:** Bahadar S Bhatia, John F Morlese, Sarah Yusuf, Yiting Xie, Bob Schallhorn, David Gruen

**Affiliations:** Directorate of Diagnostic Radiology, Sandwell & West Birmingham NHS Trust, Lyndon, West Bromwich B71 4HJ, United Kingdom; Space Research Centre, Physics & Astronomy, University of Leicester, 92 Corporation Road, Leicester LE4 5SP, United Kingdom; Directorate of Diagnostic Radiology, Sandwell & West Birmingham NHS Trust, Lyndon, West Bromwich B71 4HJ, United Kingdom; Directorate of Diagnostic Radiology, Sandwell & West Birmingham NHS Trust, Lyndon, West Bromwich B71 4HJ, United Kingdom; Merge, Merative (Formerly, IBM Watson Health Imaging), Ann Arbor, Michigan, MI 48108, United States; Merge, Merative (Formerly, IBM Watson Health Imaging), Ann Arbor, Michigan, MI 48108, United States; Jefferson Radiology and Radiology Partners, 111 Founders Plaza, East Hartford, Connecticut CT 06108, United States

**Keywords:** aortic dilatation, pulmonary emphysema, pulmonary nodule, pulmonary embolism, rib fractures, pneumothorax, deep learning, natural language processing

## Abstract

**Objectives:**

This diagnostic study assessed the accuracy of radiologists retrospectively, using the deep learning and natural language processing chest algorithms implemented in Clinical Review version 3.2 for: pneumothorax, rib fractures in digital chest X-ray radiographs (CXR); aortic aneurysm, pulmonary nodules, emphysema, and pulmonary embolism in CT images.

**Methods:**

The study design was double-blind (artificial intelligence [AI] algorithms and humans), retrospective, non-interventional, and at a single NHS Trust. Adult patients (≥18 years old) scheduled for CXR and CT were invited to enroll as participants through an opt-out process. Reports and images were de-identified, processed retrospectively, and AI-flagged discrepant findings were assigned to two lead radiologists, each blinded to patient identifiers and original radiologist. The radiologist’s findings for each clinical condition were tallied as a verified discrepancy (true positive) or not (false positive).

**Results:**

The missed findings were: 0.02% rib fractures, 0.51% aortic aneurysm, 0.32% pulmonary nodules, 0.92% emphysema, and 0.28% pulmonary embolism. The positive predictive values (PPVs) were: pneumothorax (0%), rib fractures (5.6%), aortic dilatation (43.2%), pulmonary emphysema (46.0%), pulmonary embolus (11.5%), and pulmonary nodules (9.2%). The PPV for pneumothorax was nil owing to lack of available studies that were analysed for outpatient activity.

**Conclusions:**

The number of missed findings was far less than generally predicted. The chest algorithms deployed retrospectively were a useful quality tool and AI augmented the radiologists’ workflow.

**Advances in knowledge:**

The diagnostic accuracy of our radiologists generated missed findings of 0.02% for rib fractures CXR, 0.51% for aortic dilatation, 0.32% for pulmonary nodule, 0.92% for pulmonary emphysema, and 0.28% for pulmonary embolism for CT studies, all retrospectively evaluated with AI used as a quality tool to flag potential missed findings. It is important to account for prevalence of these chest conditions in clinical context and use appropriate clinical thresholds for decision-making, not relying solely on AI.

## Introduction

A substantial amount of digital X-ray radiographs and CT scans are performed as standard-of-care in many acute NHS Trusts. It has been estimated that the day-to-day discrepancy rate of missed findings is between 3% and 5%.^1^^,^^2^ This is set amongst an ever-increasing pressure on the radiologists’ productivity, which may result in increased risk of missed findings.^3^ Within this context, it is important to maintain the quality of the radiologist’s report findings.

The use of artificial intelligence (AI) in radiology image interpretation is an exciting reality. There are a range of AI-assisted commercially developed algorithms to support reading of chest X-ray radiographs (CXR, DICOM modality DX) and chest CT scans, which are positioned as an adjunct to the radiologist’s interpretation. However, it is important to baseline the diagnostic accuracy of radiologists to target the implementation of AI-assisted solutions. Here, AI can also be used as a quality tool using retrospective images and reports. There are other aspects to the use of AI-assisted reporting including the time taken, earlier detection, reduction in out-sourcing, and improving the in-hospital radiology workflows.

We believe it is important to baseline the diagnostic accuracy of chest X-ray and chest CT using AI. To our understanding, this study spans the gap in knowledge for using AI as a quality tool in order to target the implementation of AI-assisted solutions. In this study, we used Clinical Review version 3.2 (CR3.2) as a quality tool which served to improve radiological practice.

There are other algorithms for chest X-ray,^4–6^ which are positioned as AI-assisted commercial solutions and evaluated retrospective datasets with receiver operating characteristic area under the curve (AUC) findings. One AI-assisted chest X-ray study^7^ reported missed abnormalities detection yield and false positive rate of 1.2% and 0.97%, respectively. Similarly, algorithms for chest CT showed AI-assisted identification of pulmonary nodules^8^ and incidental pulmonary embolism.^9^

## Objectives

To assess the efficacy of AI (index test) to support this quality workflow in an acute NHS Trust, we selected a sponsor to collaboratively assess their chest algorithms implemented in CR3.2. This had proprietary image deep learning (DL) and natural language processing (NLP) algorithms for pneumothorax and rib fractures in CXR images, aortic aneurysm, pulmonary nodules, emphysema, and pulmonary embolism CT images.

For each clinical condition, the following were recorded:

1. The total number of AI-flagged findings.
2. The proportion of these verified findings to the total number of AI-flagged findings.

Our hypothesis was that the number of missed findings for all chest conditions was lower than 3% to 5%.^1^^,^^2^

The null and alternate hypotheses respectively were therefore:


$$\begin{array}{c} H0:\;\text{pcr}3=\text{prad}\\ \text{HA}:\;\text{pcr}3<\text{prad} \end{array}$$

where:

pcr3 = AI-flagged missed findings verified by the radiologist per total number of readings;

prad = *expected* missed findings per total number of readings.

## Methods

### Study design

The study design was double-blind (AI algorithms and humans), retrospective, non-interventional, and single NHS centre. Both the algorithms and radiologists were “blinded” using deidentified study data. Study and report data were de-identified on secured hospital computers and network using open-source tools: the Radiological Society of North America Clinical Trials Processor (CTP)^10^ and National Institute of Health-National Library of Medicine Scrubber,^11^ respectively.

The de-identified AI findings were assigned to the lead radiologists for their review by simple randomization. This was ascribed to the random order studies and their reports arrived for processing by the AI algorithms, with processing triggered by the arrival of the reports. The processing list of deidentified flagged was simply randomized by the clinical scientist to avoid selection bias and sent to both lead radiologists.

A record of the link between the patient and the de-identified clinical data was retained solely by the lead clinical scientist on secured hospital computers. This coding key was retained in case of need to re-identify a patient where a significant missed finding was identified by the algorithms and verified by the two lead radiologists, whereupon the established NHS Trust standard operating procedure could be followed to notify the patient’s clinical team.

The original study data resided on the hospital’s imaging archive (Merative PACS) and reports on the hospital’s radiology information system (Magentus CRIS), and readily available for this retrospective study. The study was sponsored by IBM Watson Health Imaging within a single NHS centre.

#### Algorithms 

At 98% specificity, the “out-of-box” sensitivity within the DL algorithms was set as: 50% for rib fractures, pulmonary nodules, and pulmonary emphysema; 60% for pulmonary embolus; 65% for pneumothorax and aortic dilatations. At 98% specificity, the “out-of-box” sensitivity within the NLP algorithms ranged between 80% and 90%. Algorithm training was performed by experienced US radiologists using a set of ground truth studies (DICOM images and HL7 reports) of ∼40 000 studies from the United States. The algorithms were agnostic to the radiographic equipment manufacturer.

Table 1 shows the testing datasets used for imaging studies alone, reports alone, and combined imaging studies and reports.

**Table 1.** Testing dataset from multiple sources in the United States.

Testing datasets	Aortic dilatation	Pulmonary emphysema	Pulmonary nodules	Pulmonary embolism	Pneumothorax	Rib fractures
Imaging studies alone	2500	980	2400	1900	15 000	4000
Reports alone	1666	1281	1666	1666	1666	1666
Combined Imaging studies and reports	20 000	20 000	20 000	20 000	20 000	20 000

No ethnicity information was provided but testing datasets were deemed free from ethnicity bias.

### Participants

Adult patients (≥18 years old) who were scheduled to attend their CXR and/or CT imaging of the chest at an NHS Trust in the UK (NHST) were invited to enroll as participants through an opt-out process. NHST is a busy acute Trust with Siemens AG (Erlangen, Germany) Somatom Flash, AS, Perspective, Drive and Definition Edge CT scanners, and Ysio Max CXR units.

The enrolled participants were recruited as outpatients through informed written consent using an automated enrolment and opt-out procedure.^12^ Patients were provided with the research information, and a privacy notice. We incorporated their prior studies. where they had previous episodes in the Emergency Department (ED) or as in-patients within 3 years of the date of imaging attendance. Adult individuals who were unable to understand or consent on their own behalf were excluded from the study.

The opt-out mechanism for ethical consent aligned with the UK Common Law Duty of Confidentiality. The appointment letter included a written tear-off slip for opt-out should an individual chose not to participate. Written patient information was translated from English into the Trust’s five most common minority languages (Polish, Romanian, Panjabi, Urdu, and Bengali).

If the patient wished to opt-put (before enrolment) or during the study (withdrawal), then the system administrator used a database script to remove the patient and their associated records from the research database.

This enrolment process did not affect the participants’ standard-of-care imaging. Secondly, there was no bias introduced in the data selection, and the potential participants had at least two weeks to read the research information in their appointment letter prior to attendance.

### Procedures

A participant’s current images at attendance as standard-of-care were randomly assigned to a local radiologist for reporting. Images and corresponding reports which matched the clinical conditions processed by the algorithms, were identified by the lead clinical scientist, and sent to an on-premise server for de-identification. Non posterior-anterior chest CXR images were excluded. We chose to collect the previous three years’ images and reports on attendance as well, because radiologists can refer to prior findings within this period.

The de-identified images and de-identified reports were received by the CR3.2 inbox, hosted in IBM Cloud.

Two senior UK radiologists of >15 years’ experience [S.Y. (22) and J.F.M. (19)] acted as reference standards so that the total number of missed findings could be ascertained. A Fellowship-trained US radiologist of >25 years’ experience [D.G. (26)] acted as an external arbitrator.

Each radiologist was asked to review each AI-flagged discrepant finding independently and were blinded to the original patient identifier, age, biological sex, and original radiologist.

The de-identified AI-flagged discrepancy findings were presented as textual information, describing the quadrant with the finding, and type of chest condition. This was designed to mitigate cognitive bias.

For each clinical condition flagged by the algorithms, the radiologist’s findings within the image and within the report were tallied as a verified discrepancy (true positive) or not (false positive).

If both radiologists confirmed the potential finding AI-flagged and determined that the observation should be added to the report, the finding was considered as a verified finding. In the event of a difference of opinion between these radiologists, the external radiologist reviewed the case, advising on a definitive radiological determination by consensus. This reference standard was the best available method for verifying the AI-flagged discrepancy findings as missed.

### Ethical statement

This research study was approved by the London—Southeast Research Ethics Committee, REC reference 20/PR/0091, Protocol Number IASO-2303, IRAS project ID #278884, and closed to recruitment in March 2022. The research used the public interest task to manage the data. The legal basis for processing was GDPR Article 6(1)(e) and Article 9(2)(j).

### Statistical analyses

AUC analyses were not performed as this is only relevant to the testing stage of AI algorithms, not requiring subjective assessment. The AUC provides a global view of sensitivity and specificity, independent of prevalence.^13^ Instead, as the radiological context was known, the main purpose of the study is to obtain precise estimates and corresponding confidence intervals (CIs) of the following:

- Positive predictive value (PPV)—This was expressed as the proportion of verified findings divided by the total identified potential finding, and with the corresponding 95% Agresti-Coull CI.
- Rate of verified findings identified by CR3.2—This was expressed as the number of verified findings per 1000 readings, along with a 95% Poisson-based CI.

Therefore, the following were collected:

- Total number of studies processed by CR3.2
- Total number of AI-flagged findings
- Total number of verified findings identified by the radiologists

#### Sample size considerations

##### Positive predictive value

The sample size chosen was based on the precision around the PPV estimate resulting from the study data. This PPV was anticipated to be ∼20% based on radiologists’ feedback during development of the algorithms. For example, for every one pulmonary embolism that was correctly identified as a potential finding that should be added to the radiology report (ie, true positives), we expected at most 4 that are incorrectly identified (ie, false positives).

Based on an initial sample size of 10 000, for each clinical condition, the data should contain at least five verified findings and have ∼25 total potential findings identified. By using the Agresti-Coull CI for a single proportion, the sample size produces a two-sided 95% CI with a width equal to 0.31. For all six clinical conditions processed by the algorithms, the sample size produces a two-sided 95% Agresti-Coull CI with a width equal to 0.13. Both analyses were performed using https://epitools.ausvet.com.au/ciproportion.

##### Verified findings per 1000 readings

The rate of verified findings was calculated as the quotient of the number of verified missed findings to the total number of cases per 1000 readings.

In addition, a 95% Poisson-based CI for this estimate was calculated.^14^

The estimated CIs based on the Poisson distribution for the number of verified findings per 1000 readings are shown below in Table 2.

**Table 2.** Verified findings per 1000 readings (95% confidence interval).

Number of true missed	Number of readings	**Verified findings per 1000 readings (95% confidence interval)** ^a^
10	10 000	1.0 (0.48, 1.84)
20	10 000	2.0 (1.22, 3.09)
30	10 000	3.0 (2.03, 4.29)

a Based on the Poisson distribution.

##### Hypothesis testing

This was tested using a Pearson’s chi-square test for the difference between two independent proportions, and the significance level was 0.05.

## Results

**Figure 1.**
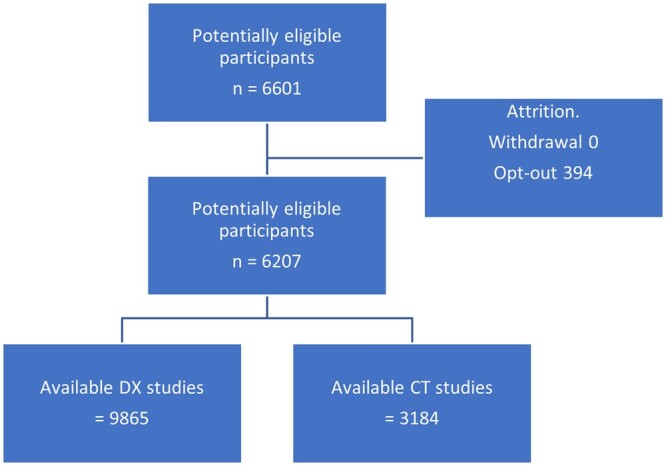
Flow of participants in this study.

From the start of the study December 4, 2020 to March 31, 2022, there were 12 650 studies with 6207 participants who had consented. The attrition through opt-out was 394 and withdrawal zero, summarized in Figure 1.

The enrolled participants reflected the ethnicity distribution in the regional population, ∼50% White and 50% minority ethnic groups. There were no adverse events. There were 9865 CXR and 3184 CT studies, total studies 13 049 available for analysis shown in Table 3. No images were excluded owing to poor radiographic image quality. There was no missing data nor indeterminate data, with radiology reports generated within 3 weeks of image acquisition.

**Table 3.** Outcomes of analysed CXR and CT images.

Finding	Available studies	Processed studies	AI-flagged findings	Verified discrepancies	Positive predictive value/%
Pneumothorax	9865	9657	33	0	0.0
Rib fractures		9864	36	2	5.6
Aortic dilatation	3184	3142	37	16	43.2
Pulmonary nodules		3142	109	10	9.2
Pulmonary emphysema		3142	63	29	46.0
Pulmonary embolism		2131	52	6	11.5
Total	13 049	9864 CXR 3142 CT	330	63	19.1

Processed studies are those where images are available to be analysed by the DL algorithms on receipt of the corresponding report.

The arrival of the reports at the CR3.2 gateway triggered the processing of both corresponding studies and reports. However, as shown in Table 3, not all reports had arrived in time to trigger this processing of studies during the analysis period.

The anticipated PPV was ∼20%. For the final sample size 13 006, the estimated prevalence for each condition was 0.05 × 13 006 = 650; 5% of these would be expected to result in an identified potential finding, as shown in Table 4 with a 95% Agresti-Coull CI.

**Table 4.** Anticipated positive predictive value (PPV) and 95% Agresti-Coull confidence intervals (CIs).

1 – α	TP	FP	Total	PPV	Lower CI	Upper CI
0.95	7	26	33	0.20	0.1038	0.3805

Thus, one would expect 33 potential findings per condition and 7 expected verified findings per condition and should be compared with Table 3.

Table 5 shows the verified findings per 1000 readings (95% CI).

**Table 5.** Verified findings per 1000 readings (95% confidence interval).

Number of true missed	Number of readings	Verified findings per 1000 readings (95% confidence interval)
10	13 0006	0.77 (0.37, 1.41)
20	13 0006	1.54 (0.94, 2.38)
30	13 0006	2.31 (1.56, 3.29)

With the observed verified findings per condition, Table 3, and 7 expected verified findings per condition (Table 4), the Pearson’s chi-square test statistic derived *P* < .0005 which was significant. The alternate hypothesis was retained.

**Figure 2.**
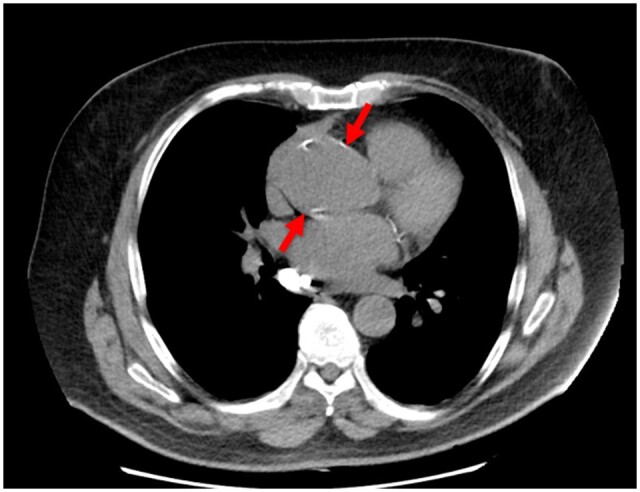
Axial view of missed findings for aortic dilatation (arrows) on a non-contrast CT chest.

**Figure 3.**
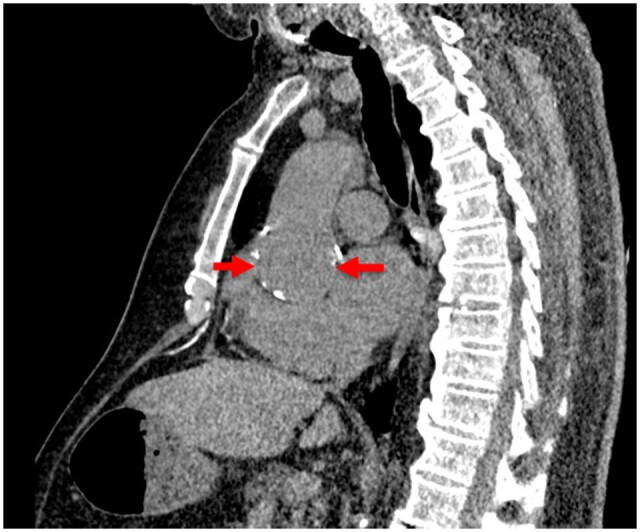
Sagittal missed findings for aortic dilatation (arrows) on a non-contrast CT chest.

**Figure 4.**
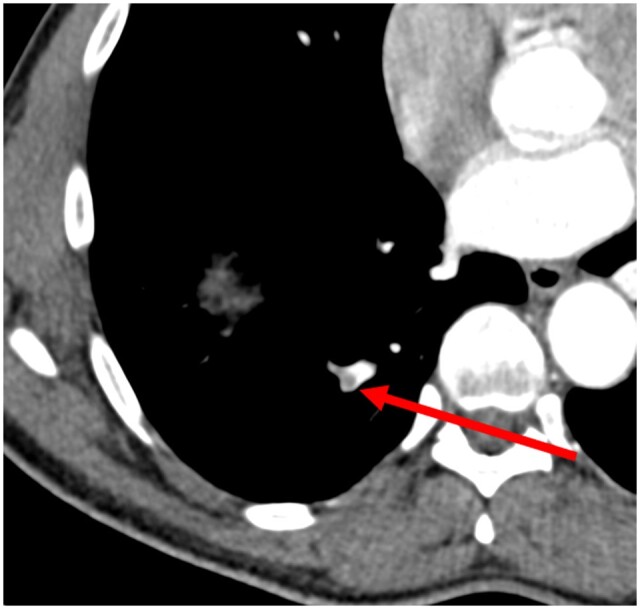
Axial view of missed findings for a pulmonary embolism (arrow) on a contrast-enhanced CT chest.

The following images show a missed finding of aortic dilatation (Figure 2 [axial] and Figure 3 [sagittal]). A missed finding of pulmonary embolism is shown in Figure 4. Both missed findings are discussed in the following section.

## Discussion

This study was a real-world evaluation of the CR3.2 chest DL and NLP algorithms within a busy acute hospital. Yet despite the ever-increasing pressure on the radiologists’ productivity, the local radiologists generated a low number of missed findings for the specified clinical conditions [0.02% for rib fractures CXR, 0.51% for aortic dilatation, 0.32% for pulmonary nodule, 0.92% for pulmonary emphysema, and 0.28% for pulmonary embolism for CT] for studies evaluated retrospectively, demonstrated in Table 3 compared to the expected missed findings.^1^^,^^2^

### CXR

The PPV for rib fractures was 5.6%, Table 3. The rib fractures missed by radiologist would be important in the acute setting and AI-assisted reporting would reduce the error.

The PPV for pneumothorax was 0%, Table 3 owing to lack of available studies that were analysed for outpatient activity; any participants with prior in-patient activity for pneumothorax would likely be treated early in their hospital episode.

Comparison of these CXR findings with published literature is not straight-forward as AUC were used.^4–6^

### CT

The PPV for aortic dilatation was moderate at 43.2%, Table 3; noting that for aortic dilatations, there is no international clinical standard for prescribed measurements away from the aortic root.^15^ In this context, the image algorithms for aortic dilatation performed as expected,^16^ with the findings reflected variations in the radiologist’s measurements. An example of this measurement uncertainty is shown in Figures 2 and 3. Any findings of minor significance were referred for cardiology review.

The PPV for pulmonary emphysema was moderate at 46.0%, with some AI-flagged findings relating to surgical emphysema or bullous disease.

The PPV for pulmonary nodules 9.2%, Table 3 was consistent with the expected number of verified discrepancies and reflected the 98% specificity and 50% sensitivity in the image algorithms. The image detection algorithms were confounded by the similarity between pulmonary nodules and their opacities caused by pneumonia, such as consolidation and ground-glass opacities.^17^ In this context, the radiologist could use other information such as patient symptoms and history, and the distribution of opacities to decide. Secondly, the low PPVs for pulmonary nodules were also caused by variations in the language used to describe pulmonary nodules in the radiologist report, which the NLP algorithms did not recognize. These may be described in the radiology reports for example, as lesions, soft tissue lesions, pulmonary nodularity, and pulmonary metastases depending on the clinical context. The outcome, therefore, was a lack of correlation between the NLP algorithm vocabulary bank, and the inconsistencies in terminology used by individual radiologists to report these features. Sixty-five out of 109 potential discrepancies, Table 3, were due to missed NLP findings. This may strengthen the case for standardization of terminology in the radiologist report. Similar algorithms for chest CT^8^ showed AI-assisted identification of pulmonary nodules with a sensitivity 0.74 with low specificity (one false positive per scan).

Although the number of expected verified discrepancies are near that predicted for pulmonary embolus, the PPV for pulmonary embolus at 11.5%, Table 3, was much lower than expected. This may likely be due to sub-segmental pulmonary embolus or due to non-optimized protocol for the pulmonary arteries as non-CT Pulmonary Angiogram (non-CTPA) studies were also processed by the algorithms; there may be flow artefacts or perivascular infiltrates rendering the image algorithms to struggle.^18^^,^^19^ This was reflected by the 50% sensitivity at 98% specificity, for which there was a potential risk of over-diagnosis of false positives. Similar algorithms for chest CTPA^9^ showed AI-assisted identification of incidental pulmonary embolism reduced the miss rate from 44.8% (without AI) to 2.6% which was remarkable compared to the static pulmonary embolism algorithm used in our study.

The alternate hypothesis that the number of missed findings for each chest conditions (apart from pneumothorax) was lower than 3% to 5%^1^^,^^2^ was confirmed.

A potential limitation of our study was it was a single centre. However, this was mitigated as all findings were reviewed by the external radiologist mitigating local bias. A further study using US data and local US radiologists showed similar findings (IBM Watson Health Imaging, proprietary). Although all radiologists had some differing opinions in some radiological evaluations, a consensus was achieved on the findings presented in Table 3.

The UK radiologists were typical of those trained through academic, clinical, and radiological placements so this is not a limiting factor, although it would be preferable in future to have a multicentre study.

The DL and NLP algorithms were proprietary, so it was not possible generalize these findings for other commercial algorithms. There were no clinically significant missed findings. There is potential to improve radiological outcomes by improving the consistency of reporting text (used by NLP algorithms) and regular peer review using the AI quality tool. Missed findings are likely to originate from time pressures, visual fatigue, inattentional blindness, and cognitive bias. The presentation of AI-flagged findings should be limited to text and the identified region, either as a quadrant or region-of-interest. Future research into cognitive bias and how the radiologist reports each CXR and CT is warranted.

Although this study investigated the PPVs and their corresponding CIs, the impact of false alarm, false positive events can increase the burden on the radiologist’s decision-making, productivity, and decrease confidence in the use of AI as a quality tool. So it is important to determine the optimal operating thresholds for the algorithms prior to clinical deployment, and then again to verify during the deployment and throughout the life cycle of AI-assisted solutions.

It would be interesting to assess the performance of the algorithms as a prospective study and multicentre where the DL algorithms could be used as a reporting check or as an inline triage to identify CXR^20^ and CT studies with no significant findings. However, a radiologist, the human-in-the-loop should always review the studies as they would be legally responsible.

## Conclusions

We have shown that our real-world evaluation was important to perform, validating the high diagnostic accuracy of our local radiologists. We have demonstrated the performance of the CR3.2 DL and NLP algorithms, and we believe that by sharing our experience this helps support further developments of these types of algorithms. Future research should consider improving the algorithms for the detection of pulmonary embolus and the stratification of pulmonary nodules. The intended use of AI as a quality tool augments the radiologist’s armoury, maintaining reporting quality.
